# Ameliorative Effect of a *Chenopodium formosanum*-Derived Peptide on Diabetic Wound Healing and Fibroblast Dysfunction

**DOI:** 10.3390/ijms27219541

**Published:** 2026-10-25

**Authors:** Chih-Yuan Ko, Kuan-Chen Cheng, Yi-Ling Lin, Shin-Ping Lin, Yi-Chen Chen, Chung-Hsin Wu, Thi Kim Ngan Nguyen, Yi-Ping Chuang, Ting-Hua Lai, Chen-Wen Lu, Chen-Che Hsieh, Szu-Chuan Shen

**Affiliations:** 1 Department of Clinical Nutrition, The Second Affiliated Hospital of Fujian Medical University, Quanzhou 362000, China; 2 Institute of Biotechnology, National Taiwan University, Taipei 10617, Taiwan; kccheng@ntu.edu.tw; 3 Institute of Food Science and Technology, National Taiwan University, Taipei 10617, Taiwan; 4 Department of Medical Research, China Medical University Hospital, China Medical University, Taichung 40447, Taiwan; 5 Department of Optometry, Asia University, Taichung 41354, Taiwan; 6 Department of Animal Science and Technology, National Taiwan University, Taipei 10617, Taiwan; 7 Graduate Program of Nutrition Science, National Taiwan Normal University, Taipei 11677, Taiwan; 8 School of Food Safety, Taipei Medical University, Taipei 11031, Taiwan; 9 School of Life Science, National Taiwan Normal University, Taipei 11677, Taiwan; megawu@ntnu.edu.tw (C.-H.W.);; 10 Department of Seafood Science, National Kaohsiung University of Science and Technology, Kaohsiung 81157, Taiwan

**Keywords:** peptide, diabetes, wound healing, fibroblasts

## Abstract

Oxidative stress contributes to fibroblast dysfunction in diabetic wounds. We evaluated a previously characterized glycine-rich peptide derived from *Rhizopus oligosporus*-fermented germinated *Chenopodium formosanum* (FGP) in male diabetic *db/db* mice and NIH3T3 fibroblasts. Topical FGP (20 or 100 ppm) was assessed by wound-area measurements and histology over 14 days, whereas fibroblasts exposed to 100 mM glucose were evaluated for viability, gap closure, intracellular reactive oxygen species (ROS), type I collagen expression, and repair-associated proteins. On day 14, wound contraction reached 77.6% with 100 ppm FGP and was greater than in untreated wounds (*p* < 0.05). In fibroblasts, 50 and 100 ppm FGP reduced ROS by approximately 37% and 79%, increased type I collagen expression by approximately 80–90%, and reduced the p-p65 NF-κB/p65 NF-κB ratio by 38% and 48% and the MMP-9/TIMP-1 ratio by 61% and 66%, respectively (*p* < 0.05). Both concentrations also improved gap closure, whereas VEGF expression increased significantly only at 50 ppm. Among the concentrations tested, 100 ppm showed the most consistent overall activity and is proposed as the preferred concentration for further preclinical evaluation, although formal dose optimization remains necessary. These findings support further investigation of FGP for diabetic wound repair, while tissue-level mechanisms and long-term outcomes remain to be established.

## 1. Introduction

According to the 11th edition of the International Diabetes Federation Diabetes Atlas, approximately 589 million adults aged 20–79 years were living with diabetes in 2024, and this number is projected to reach 853 million by 2050 [1]. Diabetes mellitus is characterized by chronic hyperglycemia resulting from abnormalities in insulin secretion, insulin action, or both [2]. Diabetic foot ulcer (DFU) is a serious and complex complication associated with peripheral neuropathy, peripheral artery disease, impaired tissue repair, and recurrent injury. The lifetime risk of developing a DFU has been estimated at 19–34%, and recurrence remains common even after successful healing [3]. These substantial clinical consequences highlight the need for strategies that improve tissue repair in diabetic wounds.

Normal cutaneous wound healing proceeds through overlapping phases of hemostasis, inflammation, proliferation, and remodeling. These processes require coordinated interactions among platelets, neutrophils, macrophages, keratinocytes, endothelial cells, fibroblasts, and other stromal cells [4,5]. During the proliferative and remodeling phases, fibroblasts migrate into the wound bed, contribute to granulation tissue formation, synthesize and remodel the extracellular matrix (ECM), and produce growth factors that support angiogenesis and tissue repair. In diabetes, sustained hyperglycemia, oxidative stress, persistent inflammation, and impaired cellular responses disrupt the normal transition between healing phases, resulting in defective re-epithelialization, angiogenesis, granulation tissue formation, and ECM remodeling [4]. Recent single-cell studies of human DFUs have further demonstrated substantial alterations in the wound microenvironment and fibroblast states, supporting an important role for fibroblast dysfunction in impaired diabetic wound healing [6].

Disruption of the epidermal barrier and diabetes-associated immune dysfunction also increase the susceptibility of diabetic wounds to microbial infection. Clinically infected DFUs may involve diverse or polymicrobial pathogens, and empirical antimicrobial treatment can be complicated by differences in pathogen susceptibility, prior antimicrobial exposure, disease severity, and local resistance patterns [7]. In addition, biofilm formation can contribute to persistent or recurrent infection and reduced antimicrobial responsiveness. Sustained microbial burden and biofilm-associated inflammation may further aggravate the inflammatory and oxidative microenvironment of chronic diabetic wounds [8]. Thus, oxidative stress and persistent inflammation represent interconnected barriers to effective tissue repair in DFUs.

*Chenopodium formosanum*, also known as Taiwan quinoa, contains several bioactive constituents and has demonstrated antioxidant and cytoprotective properties [9,10,11,12]. A low-molecular-weight glycine-rich peptide derived from germinated *C. formosanum* fermented with *Rhizopus oligosporus* (FGP) was previously isolated and characterized [13]. Previous studies showed that FGP possesses antioxidant and cytoprotective activities and can modulate oxidative stress-related signaling under experimental conditions [13,14]. However, whether FGP can improve diabetic wound repair and ameliorate fibroblast dysfunction under hyperglycemic stress has not been investigated. Therefore, the present study extends the previous characterization of FGP to diabetic wound healing by evaluating its topical effects in diabetic *db/db* mice and its effects on viability, migration-associated gap closure, intracellular oxidative stress, type I collagen expression, and selected repair-associated signaling proteins in NIH3T3 fibroblasts exposed to high glucose.

## 2. Results

### 2.1. Effects of FGP on Wound Healing in Diabetic Mice

**Figure 1:**
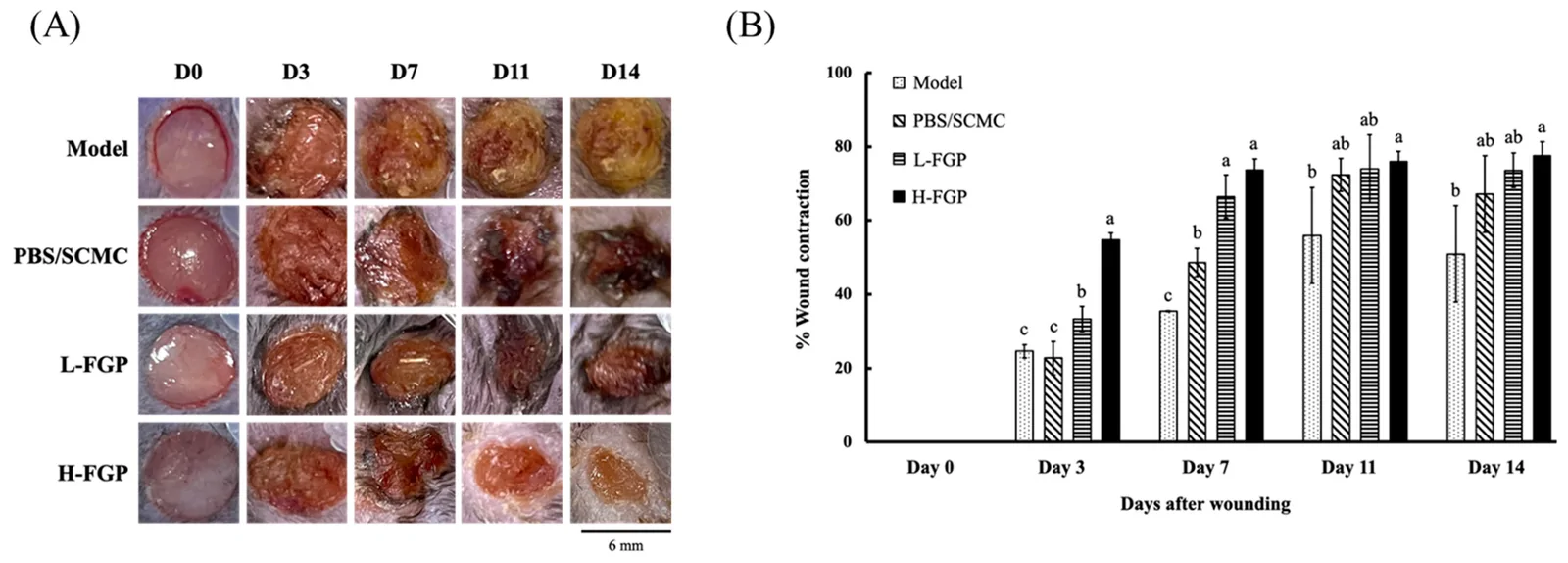
Effects of FGP on wound healing images in diabetic mice at days 0, 3, 7, 11, and 14. (**A**) Representative images. (**B**) Quantitative wound contraction. Scale bar = 6 mm. Data are presented as mean ± SEM (*n* = 3–6 mice per time point because animals were euthanized sequentially for tissue collection). Groups sharing at least one letter were not significantly different, whereas groups with no letters in common differed significantly at *p* < 0.05; one-way ANOVA followed by Duncan’s multiple range test. These comparisons are exploratory because the original analysis did not model within-mouse and repeated-measure correlations.

Wound healing was compared among untreated wounds (Model), vehicle-treated wounds receiving phosphate-buffered saline containing 2% sodium carboxymethylcellulose (PBS/SCMC), and wounds treated with 20 ppm FGP (L-FGP) or 100 ppm FGP (H-FGP). The skin wound areas of *db/db* mice in different treatment groups (Model, PBS/SCMC, L-FGP, and H-FGP) were compared (Figure 1A). After wound creation, the wounds were photographed using the same camera to document the healing progression. The L-FGP and H-FGP groups showed a higher rate of wound contraction throughout the experimental period compared to the Model and PBS/SCMC groups (Figure 1A,B). On day 3, the wound-contraction percentages in the L-FGP and H-FGP groups were 33.3% and 54.7%, respectively, which were significantly greater than those in the Model and PBS/SCMC groups (*p* < 0.05). On day 14, the wound-contraction percentage in the H-FGP group was 77.6%, which showed a significantly higher contraction rate than the Model group (*p* < 0.05) (Figure 1A,B).

### 2.2. Effects of FGP on Histological Evaluations of Skin Tissues in Diabetic Mice

**Figure 2:**
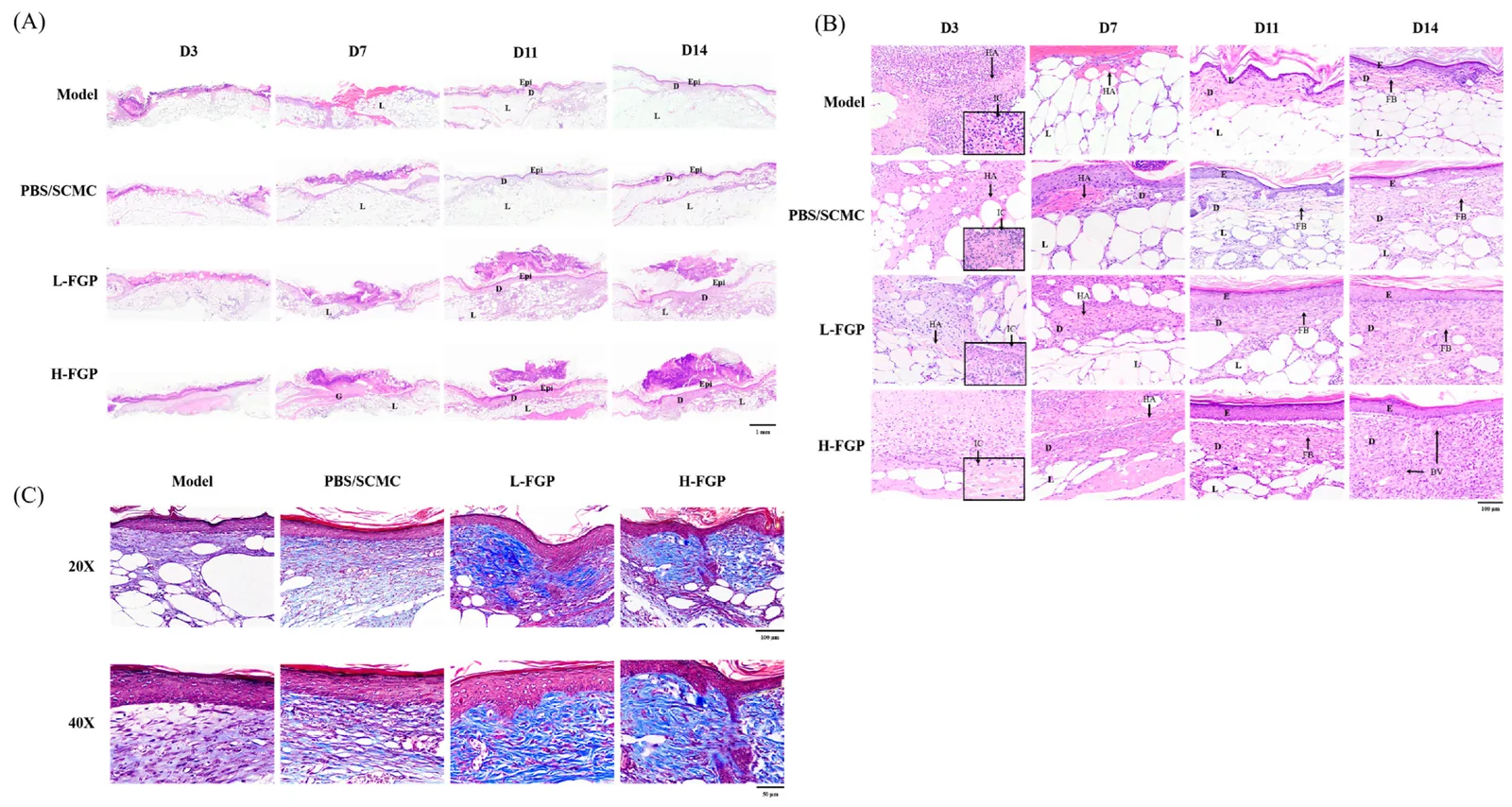
Representative histological images of wound tissues from diabetic mice treated with FGP. (**A**) Hematoxylin and eosin (H&E) staining at low magnification. (**B**) H&E staining at higher magnification. (**C**) Masson’s trichrome staining. Scale bars: A, 1 mm; B, 100 μm; C, 100 μm for the upper row and 50 μm for the lower row. Epi, epidermis; D, dermis; L, lipid layer (subcutaneous tissue); BV, blood vessel; IC, inflammatory cells; HA, hemorrhagic area; FB, fibroblast-like cells. Histological observations were qualitative.

Histological examination of wound tissues revealed qualitative differences in tissue organization among the groups (Figure 2A–C). On day 11, all groups showed formation of a new epidermal layer, as observed by hematoxylin and eosin (H&E) staining at low magnification (Figure 2A). At higher magnification, wounds in the Model group appeared to exhibit a thinner and more irregular epidermis and less organized granulation tissue, whereas the FGP-treated wounds showed a more uniform epidermal structure and denser, better-organized granulation tissue (Figure 2B). By day 14, fibroblast-like cells and vascular structures were visually more apparent in the wound tissue, particularly in the H-FGP-treated wounds (Figure 2B). Masson’s trichrome staining also appeared more prominent in the FGP-treated wounds than in the Model and PBS/SCMC wounds, suggesting greater collagen deposition (Figure 2C). These histological observations were qualitative and were not used to establish quantitative differences in epidermal or dermal thickness, collagen content, angiogenesis, or fibroblast proliferation.

### 2.3. Effects of FGP Co-Treatment on NIH3T3 Cell Viability, Intracellular ROS, Migration-Associated Gap Closure, and Type I Collagen Expression Under High-Glucose Conditions

**Figure 3:**
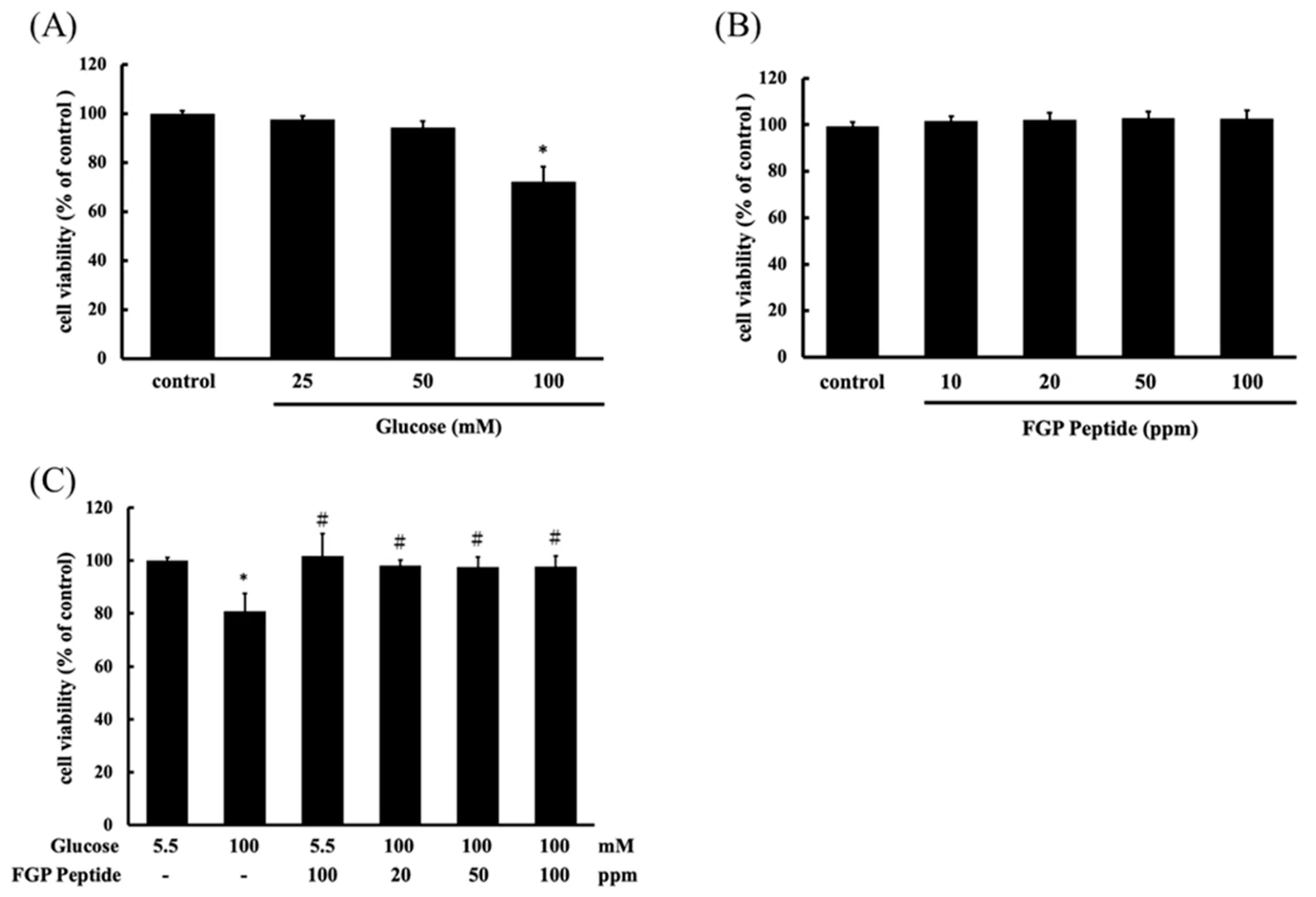
Concentration effects of (**A**) glucose, (**B**) FGP, and (**C**) FGP-glucose co-treatment on cell viability of NIH3T3 cells. Data are presented as mean ± SEM (*n* = 3 independent experiments). The stars denote *p* < 0.05 compared with the control group (5.5 mM glucose). The hashtag denotes *p* < 0.05 compared with the high-glucose group (100 mM). “−” indicates that no FGP peptide was added (0 ppm). Statistical analysis: one-way ANOVA followed by Duncan’s multiple range test.

NIH3T3 fibroblasts were treated with medium containing various concentrations of glucose for 24 h (Figure 3A). Compared with the control group (5.5 mM glucose), treatment with 100 mM glucose significantly reduced cell viability by approximately 20% (*p* < 0.05), and this concentration was used as the high-glucose condition in subsequent experiments. FGP alone at concentrations of 10, 20, 50, and 100 ppm did not significantly alter NIH3T3 fibroblast viability compared with the untreated control (*p* > 0.05) (Figure 3B). Under high-glucose conditions, co-treatment with 20, 50, and 100 ppm FGP significantly increased cell viability compared with the high-glucose group (*p* < 0.05) (Figure 3C).

**Figure 4:**
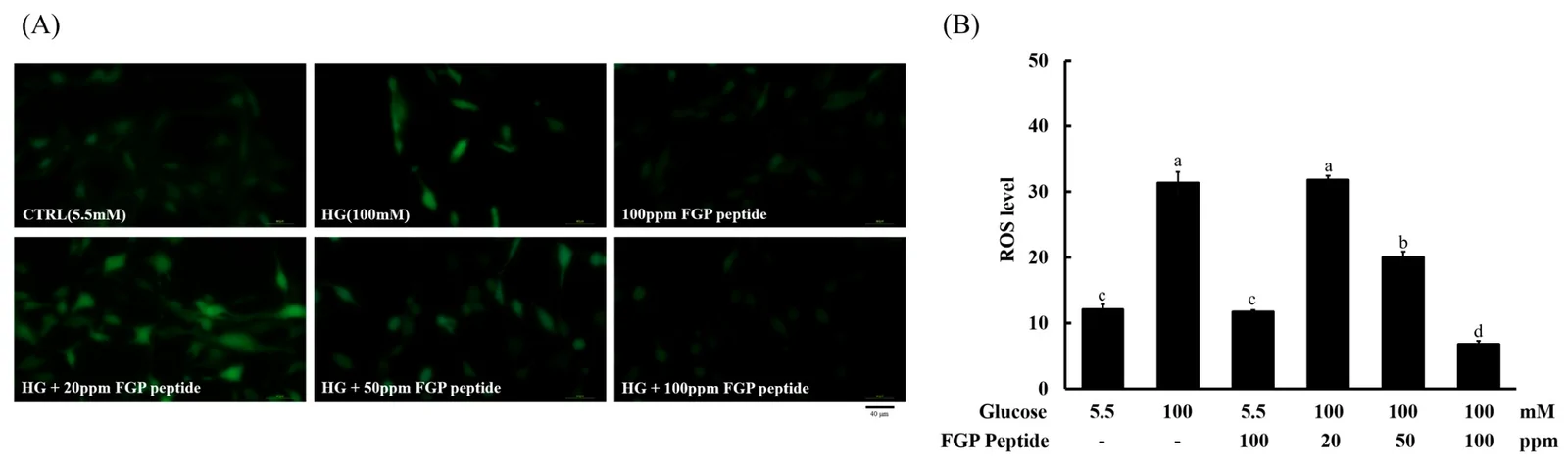
Effects of FGP on intracellular ROS levels in NIH3T3 fibroblasts under high-glucose conditions. (**A**) Representative DCFH-DA fluorescence images. Scale bar = 40 μm. (**B**) Quantitative analysis of fluorescence intensity. “−” indicates that no FGP peptide was added (0 ppm). Data are presented as mean ± SEM (*n* = 3 independent experiments). Groups sharing at least one letter were not significantly different, whereas groups with no letters in common differed significantly at *p* < 0.05; one-way ANOVA followed by Duncan’s multiple range test. Brightness and contrast were adjusted uniformly across all fluorescence panels for display only; quantification was performed using the original images.

Representative fluorescence photomicrographs of NIH3T3 fibroblasts after 24 h of treatment are shown in Figure 4A. Compared to the control group (5.5 mM glucose), high-glucose treatment resulted in a significant 2.6-fold increase in reactive oxygen species (ROS) production (*p* < 0.05) (Figure 4A,B). Co-treatment with FGP at concentrations of 50 and 100 ppm under high-glucose conditions significantly reduced ROS levels by approximately 37% and 79%, respectively, compared to the high-glucose group (*p* < 0.05).

**Figure 5:**
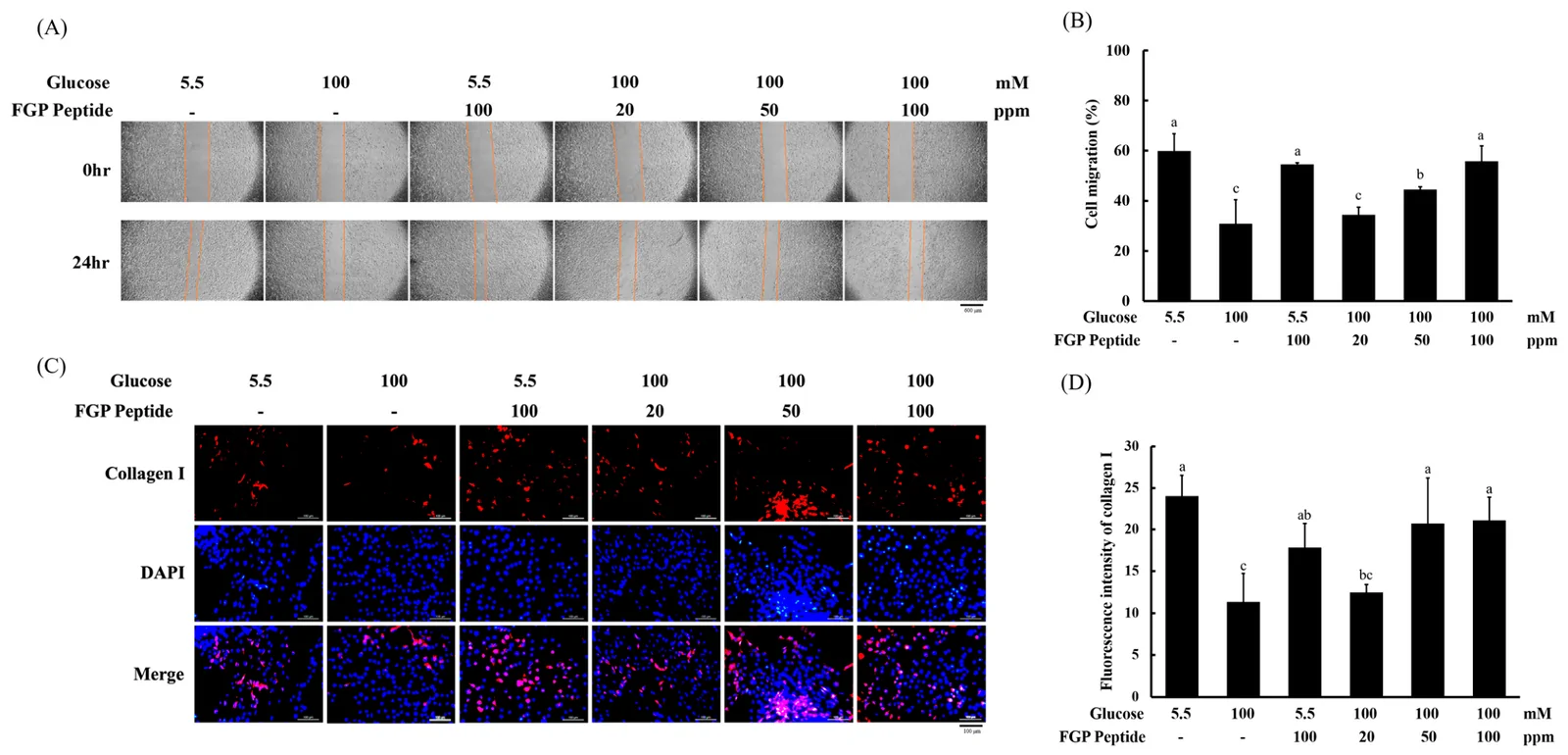
Concentration effects of FGP on migration-associated gap closure and type I collagen expression in NIH3T3 cells incubated in high-glucose medium. (**A**) Representative gap-closure images. Scale bar = 600 μm. (**B**) Quantitative analysis of gap closure. (**C**) Collagen I immunofluorescence images. Scale bar = 100 μm. (**D**) Quantitative analysis of collagen I fluorescence intensity. “−” indicates that no FGP peptide was added (0 ppm). Data are presented as mean ± SEM (*n* = 3 independent experiments). Groups sharing at least one letter were not significantly different, whereas groups with no letters in common differed significantly at *p* < 0.05; one-way ANOVA followed by Duncan’s multiple range test. Brightness and contrast were adjusted uniformly across panels for display only; quantitative analyses were performed using the original images.

In the high-glucose group (100 mM), fibroblast gap closure was significantly reduced compared with both the control group (5.5 mM glucose) and the FGP group co-treated with 5.5 mM glucose and 100 ppm FGP (*p* < 0.05) (Figure 5A,B). Under high-glucose conditions, co-treatment with 50 and 100 ppm FGP significantly enhanced gap closure compared with the high-glucose group, whereas 20 ppm FGP did not produce a significant improvement (*p* < 0.05) (Figure 5B). Figure 5C,D illustrate the effects of FGP co-treatment on type I collagen expression in NIH3T3 fibroblasts. Type I collagen expression was significantly decreased in the high-glucose group (100 mM) compared with the control group (5.5 mM glucose) and the FGP group co-treated with 5.5 mM glucose and 100 ppm FGP (*p* < 0.05). Co-treatment with 50 and 100 ppm FGP under high-glucose conditions significantly increased type I collagen expression by approximately 80–90% compared with the high-glucose group (*p* < 0.05).

### 2.4. Effects of FGP Co-Treatment with NIH3T3 Cells in High-Glucose Medium on the Healing Pathway of Cells

**Figure 6:**
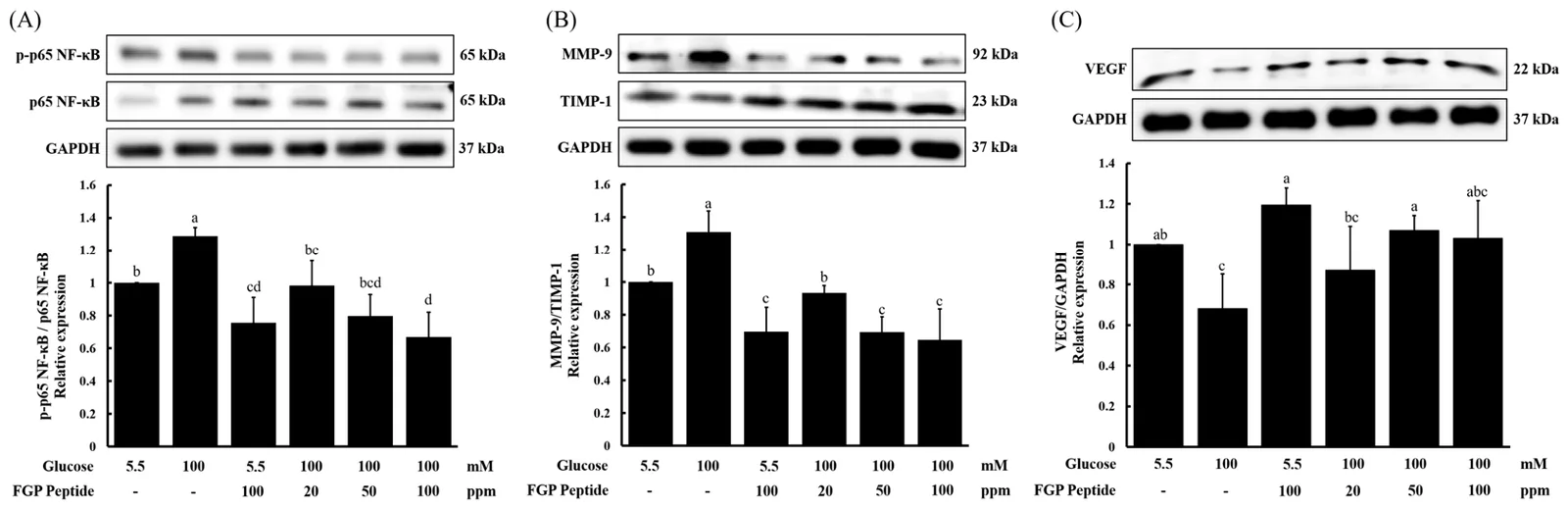
Protein expression of (**A**) p-p65 NF-κB/p65 NF-κB, (**B**) MMP-9/TIMP-1, and (**C**) VEGF in NIH3T3 cells incubated in high-glucose medium with FGP. “−” indicates that no FGP peptide was added (0 ppm). Data are presented as mean ± SEM (*n* = 3 independent experiments). Groups sharing at least one letter were not significantly different, whereas groups with no letters in common differed significantly at *p* < 0.05; one-way ANOVA followed by Duncan’s multiple range test.

Compared with the control group (5.5 mM glucose), the high-glucose group (100 mM) exhibited a significant increase in the p-p65 nuclear factor kappa B (NF-κB)/p65 NF-κB and matrix metalloproteinase 9/tissue inhibitor of metalloproteinases 1 (MMP-9/TIMP-1) protein expression ratios (*p* < 0.05), together with reduced vascular endothelial growth factor (VEGF) protein expression (Figure 6). Co-treatment with 20, 50, and 100 ppm FGP under high-glucose conditions reduced the p-p65 NF-κB/p65 NF-κB ratio by approximately 24%, 38%, and 48%, respectively, and reduced the MMP-9/TIMP-1 ratio by approximately 37%, 61%, and 66%, respectively, compared with the high-glucose group (*p* < 0.05) (Figure 6A,B). VEGF expression was significantly higher with 50 ppm FGP than in the high-glucose group, whereas the 20 and 100 ppm FGP groups were not significantly different from the high-glucose group (Figure 6C).

## 3. Discussion

This study investigated whether FGP could improve diabetic wound repair and ameliorate fibroblast dysfunction under high-glucose conditions. Topical FGP treatment was associated with greater wound contraction during the 14-day observation period in diabetic *db/db* mice, while qualitative histological examination suggested improved epidermal and granulation tissue organization and more prominent collagen staining in FGP-treated wounds. In parallel, FGP improved NIH3T3 fibroblast viability and migration-associated gap closure, reduced intracellular ROS-related fluorescence, and increased type I collagen expression under high-glucose conditions. These cellular changes were accompanied by reduced p65 NF-κB phosphorylation and a lower MMP-9/TIMP-1 expression ratio. Together, these findings provide complementary in vivo and in vitro evidence for the biological activity of FGP, although the cellular molecular changes cannot be assumed to directly mediate the wound-healing effects observed in mice.

Fibroblast dysfunction is an important feature of impaired diabetic wound repair. Previous studies have shown that fibroblasts from diabetic patients exhibit reduced migratory capacity, impaired VEGF production, and an attenuated response to hypoxia [15]. Consistent with these observations, high-glucose exposure in the present study impaired fibroblast viability, gap closure, and type I collagen expression, whereas FGP partially improved these cellular responses. These findings support a potential beneficial effect of FGP on selected fibroblast functions relevant to tissue repair. However, the gap-closure assay cannot completely distinguish cell migration from changes in cell number, and collagen immunofluorescence reflects protein abundance rather than a direct measurement of collagen synthesis. Therefore, these findings should be interpreted as changes in repair-associated cellular functions rather than direct evidence of enhanced collagen biosynthesis.

Oxidative stress and persistent inflammatory signaling are closely associated with diabetic wound dysfunction. In the present study, exposure to 100 mM glucose, which was used as a severe cellular stress condition rather than a physiological glucose concentration, increased intracellular ROS and p65 NF-κB phosphorylation, whereas FGP attenuated both responses. Previous studies have implicated NF-κB activation in prolonged inflammatory responses and impaired tissue repair under diabetic conditions [16,17]. In a diabetic rat wound model, El-Samad et al. reported that a carboxymethyl cellulose/sericin hydrogel improved antioxidant indices and re-epithelialization while reducing pro-inflammatory marker expression [18]. Because NF-κB regulates multiple inflammatory genes, these findings provide biological context for the increased p65 phosphorylation observed in our high-glucose fibroblast model. However, individual inflammatory cytokines were not measured in the present study, and the experiments did not include pharmacological inhibition, gene silencing, or other pathway-specific interventions. Accordingly, the observed reduction in p65 phosphorylation should be interpreted as an associated signaling response rather than confirmation that FGP directly targets NF-κB or establishes a complete anti-inflammatory mechanism.

Matrix metalloproteinase regulation represents another relevant component of diabetic wound repair. Excessive MMP-9 activity has been associated with poor healing of diabetic foot ulcers [19], while experimental suppression of MMP-9 has been reported to improve diabetic wound healing [20]. In the present study, FGP treatment reduced the MMP-9/TIMP-1 protein expression ratio and was accompanied by increased type I collagen expression. This pattern is consistent with a shift toward more favorable extracellular matrix regulation. However, MMP enzymatic activity, collagen degradation, and collagen turnover were not directly measured. Therefore, the observed changes in MMP-9/TIMP-1 expression should not be interpreted as direct evidence that FGP reduced collagen degradation. Similarly, VEGF expression increased at 50 ppm FGP but did not differ significantly from the high-glucose group at 20 or 100 ppm; the present data do not establish enhanced angiogenesis, particularly because angiogenic parameters were not quantitatively assessed in the wound tissue. The impaired VEGF response previously reported in diabetic fibroblasts [15] nevertheless provides a biological context for this observation.

FGP is a low-molecular-weight glycine-rich peptide derived from germinated *Chenopodium formosanum* fermented with *Rhizopus oligosporus*. Our previous studies characterized FGP and demonstrated antioxidant and cytoprotective properties under experimental conditions [13,14]. The present study extends these observations by examining FGP in the context of diabetic wound repair and high-glucose-induced fibroblast dysfunction. This represents a different application from previous studies of *C. formosanum* extracts and their larger bioactive constituents [9,10,11,12]. The current findings therefore support further investigation of FGP as a fermentation-derived peptide for wound-related applications, rather than establishing it as a clinically effective wound-healing agent.

Several limitations should be considered when interpreting these findings. First, the unsplinted dorsal excisional wound model used in male diabetic *db/db* mice does not fully reproduce the pathophysiological characteristics or healing pattern of human diabetic foot ulcers. Closure of murine dorsal wounds is substantially influenced by wound contraction, whereas human cutaneous wounds depend more heavily on granulation tissue formation and re-epithelialization [21]. Consequently, the greater wound contraction observed after FGP treatment may not directly predict improved re-epithelialization or clinical wound closure in humans and may overstate the translational relevance of the observed in vivo effect. Re-epithelialization was not quantified separately in the present study. Future studies should therefore use splinted wound models or other approaches that minimize contraction and permit direct quantitative assessment of re-epithelialization. In addition, follow-up ended on day 14; therefore, complete wound closure, scar formation, and long-term tissue remodeling were not evaluated.

Second, the original statistical analysis of wound contraction did not explicitly account for the within-animal correlation arising from multiple treatment wounds and repeated measurements over time. Accordingly, the animal comparisons should be regarded as exploratory. Histological assessment was qualitative, and epidermal or dermal thickness, collagen-positive area, inflammatory-cell infiltration, fibroblast proliferation, angiogenesis, and re-epithelialization were not quantitatively evaluated. Treatment allocation was based on fixed wound positions rather than randomization, and formal blinding was not used during treatment administration, outcome assessment, or data analysis. These design features may have introduced positional or observer bias.

Third, the 100 mM glucose condition represents a severe cellular stress model. Although this condition was selected to induce reproducible fibroblast dysfunction within the experimental period [22], the absence of an osmotic control limits the distinction between glucose-specific effects and osmotic stress. The in vitro findings were also based on a limited number of independent experiments, which may have reduced the precision of the estimates. Furthermore, tissue-level oxidative stress, inflammatory markers, and nuclear factor erythroid 2-related factor 2 (Nrf2) signaling were not assessed. Because the archived wound tissues had been stored at −20 °C for an extended period, additional molecular analyses were not performed to avoid potentially unreliable findings resulting from reduced biomolecular stability. Further studies using models that more closely reflect human wound repair, quantitative histological assessments, longer follow-up, randomized and blinded experimental procedures, and pathway-specific interventions are required to clarify the mechanisms and translational relevance of FGP in diabetic wound repair.

## 4. Materials and Methods

### 4.1. Preparation of FGP

FGP was prepared as previously described by Hsieh et al. [13] and was supplied by the laboratory of Prof. Kuan-Chen Cheng, National Taiwan University, Taipei, Taiwan. Briefly, sterilized *Chenopodium formosanum* sprouts were inoculated with *Rhizopus oligosporus* (10^6^ spores/mL, 5% *v*/*w*) and fermented for 4 days at 35 °C in a bioreactor (FS-V-SA05P, Major Science, Taipei, Taiwan). The lyophilized fermented *Chenopodium formosanum* sprouts (FCS) were defatted with n-hexane (1:3 *w*/*v*, 37 °C, 2 h). The defatted FCS was resuspended in PBS (1:10 *w*/*v*), centrifuged (15,000× *g*, 30 min, 4 °C), and the supernatant was heated at 70 °C to inactivate enzymes. The glycine-rich protein was extracted after precipitation with ammonium sulfate (90% saturation), followed by centrifugation (15,000× *g*, 30 min, 4 °C), and subsequently fractionated into <2, 2–10, and >10 kDa fractions using Vivaspin 15R and Vivaspin Turbo 15 molecular weight cut-off (MWCO) filters. The protein fractions were purified by fast protein liquid chromatography (FPLC) (ÄKTA purifier UPC 10, GE Healthcare Life Science, Salt Lake City, UT, USA) using HiTrap G-25 desalting columns (Cytiva, Uppsala, Sweden) and further separated on Superdex 30 size-exclusion chromatography (SEC) columns (prepacked Tricorn™ glass column, GE Healthcare Life Science, Salt Lake City, UT, USA). The glycine-rich peptide FGP was obtained from the purified fractions, freeze-dried, and used in subsequent experiments.

### 4.2. Animal Model

Six-week-old male diabetic *db/db* mice (C57BLKS/J Iar-+Leprdb/+Leprdb) were purchased from the National Laboratory Animal Center. After 7 days of acclimatization, six mice were included in the in vivo wound-healing experiment. All mice were housed individually at 20–22 °C and 40–70% relative humidity under a 12 h light/dark cycle, with free access to standard chow and water. All animal procedures were approved by the Institutional Animal Care and Use Committee of National Taiwan Normal University, Taipei, Taiwan (Approval No. 110041). For wound creation, mice were anesthetized by intraperitoneal administration of Zoletil 50 (0.1 mL/100 g body weight), dorsal hair was removed, and four full-thickness excisional wounds were created using a 6 mm sterile tissue punch [23,24]. FGP was dissolved in phosphate-buffered saline containing 2% sodium carboxymethylcellulose (PBS/SCMC; Sigma-Aldrich, St. Louis, MO, USA). Treatment allocation was based on fixed dorsal wound position rather than randomization: upper left, untreated Model; upper right, PBS/SCMC vehicle control (100 μL); lower left, 20 ppm FGP in PBS/SCMC (L-FGP, 100 μL); and lower right, 100 ppm FGP in PBS/SCMC (H-FGP, 100 μL). The 20 and 100 ppm concentrations were selected as lower and upper exploratory concentrations within a range that did not reduce NIH3T3 cell viability, with 100 ppm also supported by previous FGP work [13]. Carprofen (5 mg/kg body weight) was administered subcutaneously for postoperative analgesia. Day 0 was defined as the day of wound creation, and the assigned topical treatments were applied once daily. Wound measurements and photographs were obtained on days 0, 3, 7, 11, and 14. Animals were euthanized in batches at designated time points for tissue collection, resulting in a progressive reduction in the number available for serial wound assessment. Each mouse contributed one wound to each treatment condition, providing a within-animal comparison. Formal blinding was not used during treatment administration, wound assessment, histological evaluation, or data analysis. No formal prespecified inclusion or exclusion criteria were defined in the original protocol. Wound integrity was monitored throughout the experiment, and no animal was excluded because of scratching, biting, bedding adherence, or other mechanical disruption. All measurements available before each animal’s scheduled euthanasia were retained; the numbers available at the respective time points are reported in Figure 1.

### 4.3. Wound Healing Ability

For wound assessment, mice were anesthetized with 3% isoflurane. Two perpendicular wound diameters (x and y axes) were measured using an electronic digital caliper, and photographs were obtained with a size-matched reference marker for scale. Wound area was estimated using the ellipse formula (area = π × x × y/4); image-analysis software was not used for the primary wound-area calculation. Because each mouse contributed one wound to each treatment condition, one wound-level measurement per treatment condition per mouse was used at each available time point.

To calculate wound closure as the percentage of wound-area contraction, the following formula is used:

(1) $$\mathrm{Wound}\;\mathrm{contraction}\;\left(\%\right)=\;\left[\frac{\left(\mathrm{Day}\;0\;\mathrm{wound}\;\mathrm{area}-\mathrm{Day}\;t\;\mathrm{wound}\;\mathrm{area}\right)}{\mathrm{Day}\;0\;\mathrm{wound}\;\mathrm{area}}\right]\;\times 100$$

where t represents days 3, 7, 11, or 14 after wound creation.

### 4.4. Histological Analysis

Wound tissue was collected after euthanasia, rinsed with PBS, and fixed in 4% paraformaldehyde/PBS at room temperature for 2 days. Tissues were embedded in paraffin, sectioned vertically through the wound, and stained using standard hematoxylin and eosin (H&E) and Masson’s trichrome procedures. Sections were examined using a whole-slide/panoramic scanning microscope, and representative fields were captured for morphological assessment. Histological assessment was qualitative. Dermal thickness, collagen-positive area, blood-vessel density, inflammatory-cell counts, fibroblast proliferation, and angiogenesis were not quantitatively measured.

### 4.5. Cell Culture

The embryonic mouse fibroblast cell line NIH3T3 was obtained from the Bioresource Collection and Research Center (BCRC), Hsinchu, Taiwan. NIH3T3 cells were cultured in Dulbecco’s Modified Eagle Medium (DMEM) supplemented with 10% fetal bovine serum (FBS), 1% penicillin–streptomycin (Pen-Strep), and 1% L-glutamine at 37 °C in a 5% CO_2_ atmosphere.

### 4.6. Cell Viability Assay

NIH3T3 cells were seeded in 96-well plates at 1 × 10^5^ cells/mL (200 μL/well) and allowed to attach for 24 h. Cells were then exposed for 24 h to medium containing 5.5, 25, 50, or 100 mM glucose, or to FGP at 10–100 ppm as indicated. For co-treatment experiments, FGP was added under 100 mM glucose conditions. Cell viability was assessed using the Cell Counting Kit-8 (CCK-8; Dojindo Laboratories, Kamimashiki-gun, Kumamoto, Japan). After treatment, 10 μL CCK-8 reagent was added and cells were incubated for 1 h before absorbance was measured at 450 nm using an Epoch™ Microplate Spectrophotometer (BioTek Instruments, Winooski, VT, USA).

### 4.7. Cell Migration Assay

Ibidi 2-well culture inserts were used to assess migration-associated gap closure. NIH3T3 cells were prepared at 1.5 × 10^5^ cells/mL, and 70 μL of cell suspension was seeded into each chamber. After 24 h for cell attachment, the inserts were removed to generate a 500 μm cell-free gap, and 1 mL of treatment medium was added. Gap widths at 0 h (L0) and 24 h (L24) were imaged using an IX51 inverted microscope (Olympus, Tokyo, Japan) and measured using ImageJ 1.53v. Gap closure was calculated as [(L0 − L24)/L0] × 100.

### 4.8. Determination of ROS Level

NIH3T3 cells were seeded in 24-well plates at 1 × 10^5^ cells/mL and treated for 24 h with the indicated glucose and FGP conditions. After treatment, cells were washed with PBS and incubated with 200 μM 2′,7′-dichlorodihydrofluorescein diacetate (DCFH-DA), prepared by adding 10 μL of a 20 mM DCFH-DA stock solution to 990 μL of 5% FBS/PBS, for 30–40 min at 37 °C. Cells were then washed twice with PBS, and intracellular oxidant-sensitive fluorescence was examined using an IX51 inverted fluorescence microscope (Olympus, Tokyo, Japan).

### 4.9. Immunofluorescence Staining

NIH3T3 cells were seeded at 1.5 × 10^4^ cells/well in μ-Slide 8-well chambers and treated with the indicated FGP/glucose conditions for 24 h. Cells were fixed with 2% paraformaldehyde for 10–15 min, permeabilized with 0.1% Triton X-100 for 10 min, and incubated with the primary antibody against collagen I overnight at 4 °C. After PBS washes, cells were incubated with the secondary antibody for 2 h at room temperature, counterstained with 4′,6-diamidino-2-phenylindole (DAPI), and imaged using an IX51 inverted fluorescence microscope (Olympus, Tokyo, Japan).

### 4.10. Western Blotting

NIH3T3 cells were seeded in 10 cm culture dishes at 1 × 10^5^ cells/mL, allowed to attach for 24 h, and then treated with the indicated glucose and FGP conditions for 24 h. Cells were washed with PBS and lysed in 1 mL lysis buffer on ice. Protein samples were separated by sodium dodecyl sulfate–polyacrylamide gel electrophoresis (SDS-PAGE) and transferred to polyvinylidene difluoride (PVDF) membranes. After blocking, membranes were incubated overnight at 4 °C with anti-glyceraldehyde-3-phosphate dehydrogenase (GAPDH) (1:1000; Cell Signaling Technology, Danvers, MA, USA), anti-MMP-9 (1:1000; GeneTex, Irvine, CA, USA), anti-TIMP-1 (1:1000; GeneTex), anti-VEGF (1:1000; GeneTex), anti-p65 NF-κB (1:500; Cell Signaling Technology), and anti-phospho-p65 NF-κB (1:1000; Cell Signaling Technology). After washing, membranes were incubated with anti-rabbit secondary antibody (1:10,000) for 1 h at room temperature. Protein bands were visualized using enhanced chemiluminescence and imaged with a UVP Biospectrum Imaging System (UVP, Upland, CA, USA).

### 4.11. Statistical Analysis

Experimental data were analyzed using IBM SPSS Statistics 23.0 software (IBM Corp., Armonk, NY, USA). Data are presented as mean ± standard error of the mean (SEM). For the in vitro experiments, n = 3 represents three independent experiments performed on separate days. Within each independent experiment, three parallel culture plates were treated as technical replicates, and their measurements were averaged before statistical analysis. Comparisons among treatment conditions were performed using one-way analysis of variance (ANOVA), followed by Duncan’s multiple range test. A *p* value < 0.05 was considered statistically significant. Formal assessments of normality and homogeneity of variance were not documented in the original analysis; therefore, these assumptions cannot be considered formally verified. For the in vivo wound-healing experiment, treatment conditions were compared at individual time points using one-way ANOVA. Six mice were available at baseline, and the number available for serial measurements decreased after scheduled euthanasia for tissue collection. Because multiple treatment wounds were evaluated within the same mouse and wound closure was assessed repeatedly over time, these observations were not fully independent. The original analysis did not explicitly model the within-animal or repeated-measure correlations; accordingly, the animal wound-contraction comparisons should be interpreted as exploratory.

## 5. Conclusions

**Figure 7:**
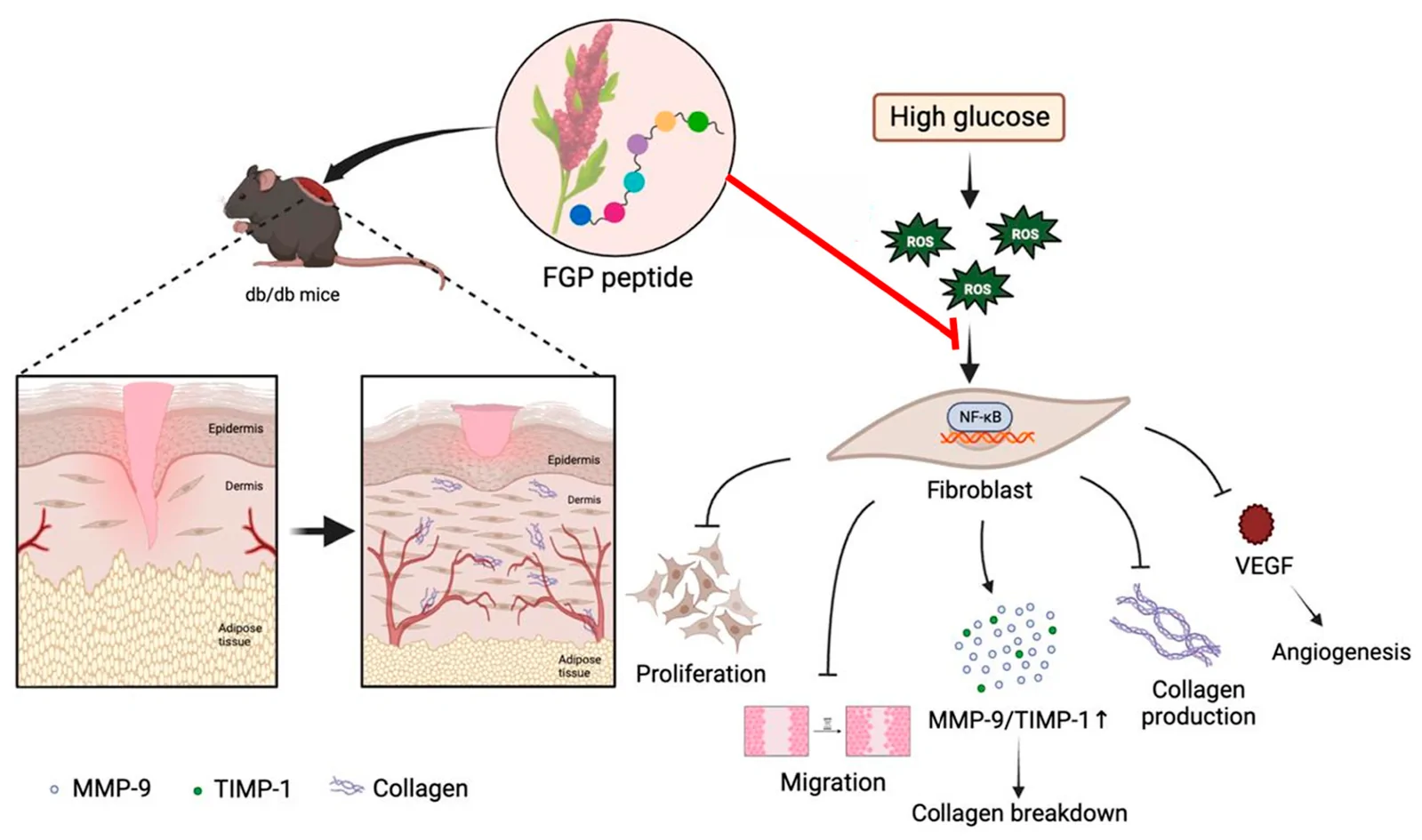
Summary of the observed effects of FGP in diabetic wounds and fibroblasts exposed to high glucose. The schematic distinguishes tissue-level observations from cellular measurements and does not establish a causal pathway linking these findings.

This study demonstrated that topical application of FGP was associated with improved wound contraction during the 14-day observation period in diabetic *db/db* mice, while qualitative histological examination suggested improved tissue organization in FGP-treated wounds. In parallel, FGP treatment under high-glucose conditions reduced intracellular ROS levels and improved NIH3T3 fibroblast viability, migration, and type I collagen expression. These cellular changes were accompanied by reduced p65 NF-κB phosphorylation and a lower MMP-9/TIMP-1 expression ratio. Collectively, these findings suggest that FGP may support selected processes involved in diabetic wound repair (Figure 7). Among the concentrations examined, 100 ppm produced the most consistent overall response and may be prioritized for subsequent preclinical dose-optimization studies. However, the observed molecular changes should be regarded as associated cellular responses rather than evidence of a confirmed causal mechanism. Further studies are needed to establish tissue-level mechanisms, long-term wound-healing outcomes, and the translational relevance of FGP-based wound dressings.

## Data Availability

The data that support the findings of this study are available from the corresponding author upon reasonable request.
